# Visual Dysfunction Predicts Cognitive Impairment and White Matter Degeneration in Parkinson's Disease

**DOI:** 10.1002/mds.28477

**Published:** 2021-01-09

**Authors:** Angeliki Zarkali, Peter McColgan, Louise‐Ann Leyland, Andrew J. Lees, Rimona S. Weil

**Affiliations:** ^1^ Dementia Research Centre University College London London United Kingdom; ^2^ Huntington's Disease Centre University College London London United Kingdom; ^3^ Reta Lila Weston Institute of Neurological Studies London United Kingdom; ^4^ Wellcome Centre for Human Neuroimaging University College London London United Kingdom; ^5^ Movement Disorders Consortium National Hospital for Neurology and Neurosurgery London United Kingdom

**Keywords:** Parkinson's disease, Parkinson's disease dementia, diffusion weighted imaging, fixel, white matter

## Abstract

**Background:**

Visual dysfunction predicts dementia in Parkinson's disease (PD), but whether this translates to structural change is not known. The objectives of this study were to identify longitudinal white matter changes in patients with Parkinson's disease and low visual function and also in those who developed mild cognitive impairment.

**Methods:**

We used fixel‐based analysis to examine longitudinal white matter change in PD. Diffusion MRI and clinical assessments were performed in 77 patients at baseline (22 low visual function/55 intact vision and 13 PD‐mild cognitive impairment/51 normal cognition) and 25 controls and again after 18 months. We compared microstructural changes in fiber density, macrostructural changes in fiber bundle cross‐section and combined fiber density and cross‐section, across white matter, adjusting for age, sex, and intracranial volume.

**Results:**

Patients with PD and visual dysfunction showed worse cognitive performance at follow‐up and were more likely to develop mild cognitive impairment compared with those with normal vision (*P* = 0.008). Parkinson's with poor visual function showed diffuse microstructural and macrostructural changes at baseline, whereas those with mild cognitive impairment showed fewer baseline changes. At follow‐up, Parkinson's with low visual function showed widespread macrostructural changes, involving the fronto‐occipital fasciculi, external capsules, and middle cerebellar peduncles bilaterally. No longitudinal change was seen in those with mild cognitive impairment at baseline or converters, even when the 2 groups were combined.

**Conclusion:**

Parkinson's patients with poor visual function show increased white matter damage over time, providing further evidence for visual function as a marker of imminent cognitive decline. © 2021 The Authors. *Movement Disorders* published by Wiley Periodicals LLC on behalf of International Parkinson and Movement Disorder Society.

The risk of dementia is markedly increased in Parkinson's disease (PD), but its onset and severity are highly heterogeneous,^1^, ^2^ making individual‐level predictions difficult and limiting timely therapeutic interventions. There is a growing body of evidence that Parkinson's patients with visual dysfunction are at a greater risk of dementia,^3^, ^4^, ^5^ but whether this translates into structural change over time is not yet known.

Animal and cell models suggest that axonal injury is an early event in Parkinson's dementia.^6^, ^7^, ^8^ In patients with PD, white matter changes are seen and can be measured using diffusion‐weighted imaging. These increase with worsening cognition and may precede gray matter atrophy.^9^, ^10^ However, white matter imaging using conventional diffusion‐tensor imaging metrics is insensitive and cannot accurately model crossing fibers, which affects a large proportion of white matter tracts.^11^

Higher‐order diffusion models, such as fixel‐based analysis (FBA) have emerged as a more sensitive, fiber‐specific framework for identifying white matter alterations.^12^ FBA quantifies changes in specific fiber populations within a voxel (“fixel”), allowing comparisons of specific tracts rather than voxel‐averaged metrics.^12^ We recently showed that FBA is more sensitive than standard techniques in identifying white matter changes in PD.^13^ Another FBA study showed macrostructural changes within the corpus callosum in patients with PD, which worsened with disease progression, but cognitive assessments were limited, and it was not designed to examine white matter changes related to cognitive progression.^14^

Here, we examined the white matter changes that evolve during the early stages of cognitive impairment in PD. We assessed patients with PD and visual dysfunction, known to be at risk of incipient dementia,^1^, ^15^, ^16^ and examined white matter alterations at baseline and after 18 months compared with PD patients with intact visual function. We also examined changes linked with PD and mild cognitive impairment (PD‐MCI) in the same cohort; we assessed white matter changes in PD‐MCI at baseline and longitudinally in PD‐MCI and patients who progressed to develop PD‐MCI. We hypothesized that patients with Parkinson's and visual dysfunction would show worsening cognitive performance and white matter degeneration at follow‐up compared with those with intact vision and that more diffuse changes would be evident in this group than in PD‐MCI and PD‐MCI converters, reflecting the greater sensitivity of poor vision as a predictor of degeneration in PD.

## 1 Methods

### 1.1 Participants

We recruited 105 patients with PD to the National Hospital, Queen Square, and 35 unaffected controls from spouses and volunteer databases. All patients satisfied the Queen Square Brain Bank criteria for PD.^17^ Participants underwent clinical assessments and brain imaging at baseline and after 18 months (visit 2).

From the total of 140 participants with imaging at baseline, 11 were unable to be scanned because of evolving contraindications or participant request, 4 withdrew, 3 were excluded because of subsequent diagnosis of atypical parkinsonian syndromes, 19 were unable to participate because of coronavirus‐related lockdown and 2 failed quality control criteria for diffusion‐weighted imaging at visit 2. This resulted in a total of 101 participants includeding 76 patients with PD and 25 controls.

Participants with PD were further classified according to their performance in 2 computer‐based visual tasks, the cats & dogs task and biological motion task (Fig. S1). Details of stimulus generation and experimental procedures were previously described.^5^, ^16^, ^18^, ^19^ Performance in these tasks is correlated with cognitive but not low‐level visual performance at baseline and worsening cognition at the 1‐year follow‐up.^5^, ^18^ We have also shown that task performance is associated with fiber‐specific white matter changes.^13^ As there is variability in individual task performance in patients with PD and controls^19^ and to capture only those participants who showed consistently low performance in both tasks, we classified as PD low visual performers participants performing worse than the group median performance on both tasks at baseline (n = 22), similar to prior work from our group.^5^, ^13^, ^20^ All others were classified as PD high visual performers (n = 54). An overall measure of visual performance was also calculated as the sum of the *Z*‐scored performance in each task (*Z*‐scored against group‐average performance, *Z*_score_ = *Z*_biolmotion_ + *Z*_cats&dogs_); see Figure S1 for more details.

All participants underwent a series of clinical and psychological assessments at both study times. Assessment of motor function was performed using the MDS‐UPDRS,^21^ general cognition using the Mini–Mental State Examination (MMSE) and Montreal Cognitive Assessment (MoCA).^22^, ^23^ Domain‐specific cognitive assessments included for attention, digit span backwards^24^ and Stroop: Naming,^25^ for executive function, Stroop Interference^25^ and category fluency,^26^ for memory: Word Recognition Task^27^ and Logical Memory,^24^ for language: Graded Naming Task^28^ and Letter fluency,^26^ and for visuospatial: Benton's Judgment of Line^29^ and Hooper Visual Organization Test.^30^ Visual acuity was assessed using LogMAR,^31^ color vision using the D15^32^ and contrast sensitivity using the Pelli‐Robson test.^33^ Smell was assessed using Sniffin' Sticks.^34^ The presence of visual hallucinations was assessed using question 2 of the UPDRS, “Over the past week have you seen, heard, smelled or felt things that were not really there?” and further information on frequency and severity of hallucinations was collected using the University of Miami Parkinson's Disease Hallucinations Questionnaire.^35^ Mood was assessed using the Hospital Anxiety and Depression Scale (HADS)^36^ and sleep using the REM Sleep Behavior Disorder Questionnaire.^37^ Levodopa‐equivalence dose score was calculated for PD participants.^38^

All participants had a MoCA score ≥ 26 at baseline, above the cutoff for Parkinson's dementia of the Movement Disorder Society Task Force criteria.^39^ PD with mild cognitive impairment (PD‐MCI) was defined as impaired performance (<1.5 SD of control performance) on at least 2 domain neuropsychological tests.^40^ We examined both PD participants with MCI at baseline (n = 13, of whom 9 were low visual performers) and those who had normal baseline cognition but developed MCI at visit 2 (n = 13, of whom 4 were low visual performers), defined here as PD‐MCI (n = 26), to capture the group at risk of declining cognitive function. We also performed subanalyses of each of these groups separately to test whether established PD‐MCI or very early changes in the converters might be driving any changes we found. All other PD participants were classified as PD with normal cognition (PD‐NC, n = 50). A composite cognitive *Z* score (averaged across the 5 individual cognitive domains) was also computed.^41^

### 1.2 MRI Data Acquisition and Processing

MRI data were acquired on the same 3 T Siemens Magnetom Prisma scanner (Siemens) with a 64‐channel head coil. Parameters for diffusion‐weighted imaging (DWI) acquisition were: b = 50 s/mm^2^/17 directions, b = 300 s/mm^2^/8 directions, b = 1000 s/mm^2^/64 directions, b = 2000 s/mm^2^/64 directions, 2 × 2 × 2 mm isotropic voxels; TE = 3260 milliseconds; TR = 58 milliseconds; 72 slices; 2‐mm thickness; acceleration factor = 2. Parameters for magnetization prepared rapid acquisition gradient echo used to compute intracranial volume in SPM12 were: 1 × 1 × 1 mm voxel, TE = 3.34 milliseconds; TR = 2530 milliseconds; flip angle = 7°.

Prior to processing, all volumes of raw data sets were visually inspected, and each volume evaluated for the presence of artifact; only scans with <15 volumes containing artifacts^42^ were included. DWI images that passed quality control underwent denoising,^43^ removal of ringing artifacts,^44^ eddy‐current and motion correction^45^ and bias‐field correction.^46^ To increase anatomical contrast, we up‐sampled the spatial resolution of diffusion‐weighted images to voxel size of 1.3 mm^3^ (using the mrresize command in MRtrix, which uses bspline interpolation); this is recommended for fixel‐based analysis when the acquired diffusion‐weighted imaging has a voxel size over 1.3 × 1.3 × 1.3 mm.^47^ We then performed intensity normalization across subjects. Preprocessing steps were performed using MRtrix3 (www.mrtrix.org).

### 1.3 Fixel‐Based Analysis

Fiber‐orientation distributions (FODs) for each participant were computed via multishell 3‐tissue‐constrained spherical deconvolution using the group‐average response function for each tissue type (gray matter, white matter, CSF).^48^ A group‐averaged FOD template was created at baseline for the purpose of longitudinal comparison from 30 randomly selected subjects (20 PD, 10 controls). Each participant's FOD image was registered to the template.^49^ Three fixel‐based metrics were then derived for each subject:

- *Fiber density (FD) —* sensitive within‐voxel alterations; measure of microstructural changes within tracts.^47^
- *Fiber cross‐section (FC) —* metric of relative differences in bundle cross‐section in which lower values suggest tract atrophy^12^; measure of macrostructural white matter change.
- *Combined measure of fiber density and cross‐section (FDC)* — combined metric calculated as FD multiplied by FC for each fixel and representing a combined measure of change at both micro‐ and macrostructural levels.^12^

### 1.4 Voxel‐Based Analysis

We also performed a voxel‐based analysis of fractional anisotropy (FA) and mean diffusivity (MD). Following DWI preprocessing, we derived the diffusion tensor from each participant's FOD image^50^ and calculated an FA and MD map in participant space. Each individual's maps were then transformed to template space, and voxel‐wise analysis was performed.

### 1.5 Statistical Analysis

#### 1.5.1 Demographics

Group differences in demographics and clinical characteristics were examined using independent *t* samples and analysis of variance (post hoc Tukey) for normally distributed continuous, Mann–Whitney and Kruskall–Wallis (post hoc Dunn) for nonnormally distributed, and chi‐square for categorical variables (Shapiro–Wilk test for normality); statistical significance was set at *P* < 0.05.

#### 1.5.2 Whole‐Brain Fixel‐Based Analysis

We used nonparametric permutation testing and connectivity‐based fixel enhancement (CFE)^51^ to identify significant differences in fixel‐based metrics. A tractogram with 20 million streamlines was generated using whole‐brain probabilistic tractography on the population FOD template. This was filtered to 2 million streamlines using spherical‐deconvolution informed filtering of tractograms).^52^ CFE was performed on the resulting streamlines with default smoothing parameters (C = 0.5, E = 2, H = 3), 5000 permutations, and family‐wise error (FWE) for multiple comparisons; these parameters have shown maximum sensitivity.^51^ FWE‐corrected *P* < 0.05 with cluster‐extent‐based threshold of 10 voxels was considered statistically significant.

Using baseline age, sex, and total intracranial volume as covariates, whole‐brain paired comparisons of fixel‐derived metrics were performed at baseline between (1) patients with PD and controls, (2) PD low versus PD high visual performers, and (3) PD‐MCI versus PD‐NC. Additional comparisons included correlation with MoCA and 2 clinically derived dementia risk scores.^53^, ^54^ To implement a longitudinal design matrix for statistical analysis, we subtracted each baseline image from the visit 2 image, as previously described.^55^ Whole‐brain statistical analysis was then performed on these difference images, covarying for baseline age, sex, and total intracranial volume. An additional analysis was performed using age, sex, total intracranial volume, and years spent in education as covariates. Whole‐brain FBA refers to the comparison of all white matter fixels within the brain using the John Hopkins University atlas.^14^, ^56^

#### 1.5.3 Whole‐Brain Voxel‐Based Analysis

Voxel‐based analysis was performed using threshold‐free cluster enhancement with default parameters (dh = 0.1, E = 0.5, H = 2)^57^ across the white matter (as in FBA), same comparisons and covariates, with FWE‐corrected *P* < 0.05 considered statistically significant.

#### 1.5.4 Tract of Interest Analysis

To investigate longitudinal change in selective fiber pathways within the visual system, we also performed tract‐of‐interest analyses. We selected 11 tracts involved in visual processing, similar to previous work^13^: posterior thalamic radiations, splenium, body and genu of the corpus callosum, superior longitudinal fasciculi, inferior fronto‐occipital fasciculi (includes inferior longitudinal fasciculus), and superior fronto‐occipital fasciculi. We calculated mean FC across each tract per participant; FC was chosen, as prior works showed it is the most sensitive fiber‐specific metric in PD.^13^, ^14^ Mean FC at baseline and longitudinally was compared between PD low versus PD high visual performers using a linear mixed model (age, sex, and intracranial volume as covariates), false discovery rate (FDR) corrected for the 11 tracts tested. Correlational analyses of mean tract FC with a combined cognitive score was performed using linear regression with age, sex, and intracranial volume as covariates.

## 2 Results

One hundred and one participants were included: 76 patients with PD (22 of whom were low visual performers) and 25 controls. Demographics and results of clinical assessments are seen in Table 1. PD high and PD low visual performers were well matched in baseline cognitive performance, vascular risk factors (Table S1), and MCI status, disease duration, motor severity, and levodopa‐equivalent dosage. MCI status was defined as MCI at baseline or conversion to MCI during follow‐up. In total 26 patients had PD‐MCI (13 at baseline and 13 converted), whereas the remaining 50 patients had normal cognition throughout.

**TABLE 1:** Demographics and results of clinical assessments

Characteristic	Controls, n = 25	PD high visual performers, n = 54	PD low visual performers, n = 22	Statistic
Age (years)	**67.4 (8.2)**	**63.7 (7.5)**	**69.3 (7.6)**	**F = 7.119** ***P* = 0.001** ^a^ ^,^ ^b^
Male (%)	12 (48)	26 (48.1)	15 (68.2)	χ ^2^ = 2.890 *P* = 0.089
Years of education	17.8 (2.2)	16.9 (2.9)	18.1 (2.5)	F = 1.919 *P* = 0.152
Total intracranial volume (cm^3^)	1542.9 (121.3)	1628.5 (189.1)	1657.0 (146.8)	F = 3.246 *P* = 0.043^b^ ^,^ ^c^
Vision				
Contrast sensitivity (Pelli Robson)^d^	**1.8 (0.2)**	**1.8 (0.2)**	**1.7 (0.1)**	**H = 17.377** ***P* = 0.001** ^a^ ^,^ ^c^
Acuity (LogMar)^d^	−0.1 (0.3)	−0.1 (0.2)	−0.1 (0.1)	H = 3.232 *P* = 0.199
Color vision (D15)	1.3 (2.4)	2.7 (8.9)	3.0 (4.9)	H = 1.934 *P* = 0.380
General cognition				
MCI	—	13 (24.1)	13 (59.1)	χ^2^ = 2.933 *P* = 0.087
MOCA	29.0 (1.1)	28.3 (1.9)	27.0 (2.7)	H = 8.333 *P* = 0.016^c^
MMSE	29.2 (0.9)	29.1 (1.7)	28.8 (1.2)	H = 1.534 *P* = 0.464
Mood				
HADS anxiety	3.5 (3.4)	5.4 (3.7)	6.5 (3.9)	H = 8.201 *P* = 0.017^b^ ^,^ ^c^
HADS depression	**1.2 (1.5)**	**3.2 (2.8)**	**5.7 (3.4)**	**H = 28.898** ***P* < 0.001** ^a^ ^,^ ^b^ ^,^ ^c^
Disease‐specific measures				
Years from diagnosis	—	3.8 (2.3)	4.9 (2.8)	t = −1.787 *P* = 0.078
UPDRS total score	—	**42.7 (16.3)**	**53.7 (28.0)**	**t = −2.102** ***P* = 0.039**
UPDRS motor score	—	22.4 (10.2)	25.8 (16.2)	t = −1.094 *P* = 0.278
Habitual hallucinations	—	6 (11.1)	6 (27.3)	χ^2^ = 1.976 *P* = 0.160
UM‐PDHQ (hallucination severity score)	—	0.5 (1.8)	1.1 (2.1)	t = −1.358 *P* = 0.179
LEDD	—	401.4 (211.6)	492.8 (225.5)	t = −1.653 *P* = 0.102
RBDSQ	—	4.0 (2.3)	4.5 (1.9)	t = −0.839 *P* = 0.404

All data shown are mean (SD) except sex and MCI.

Statistical comparisons shown are across the 3 groups with subscript for post hoc results, except for diseasespecific measures (comparing PD low and high visual performers). In bold are characteristics that significantly differed between PD low visual performers and PD high visual performers.

MCI, mild cognitive impairment; HADS, Hospital Anxiety and Depression Scale; MMSE, Mini–Mental State Examination; MoCA, Montreal Cognitive Assessment; UPDRS, Unified Parkinson's Disease Rating Scale; UM‐PDHQ, University of Miami Hallucinations Questionnaire; LEDD, levodopa‐equivalent dose; RBDSQ, REM Sleep Behavior Disorder Scale.

^a^ Statistically significant difference between PD high visual performers and PD low visual performers.

^b^ Statistically significant difference between PD high visual performers and controls.

^c^ Statistically significant difference between PD low visual performers and controls.

^d^ Best binocular score used; LogMAR, lower score implies better performance; Pelli Robson, higher score implies better performance.

### 2.1 Longitudinal Changes in Cognition

**FIG. 1:**
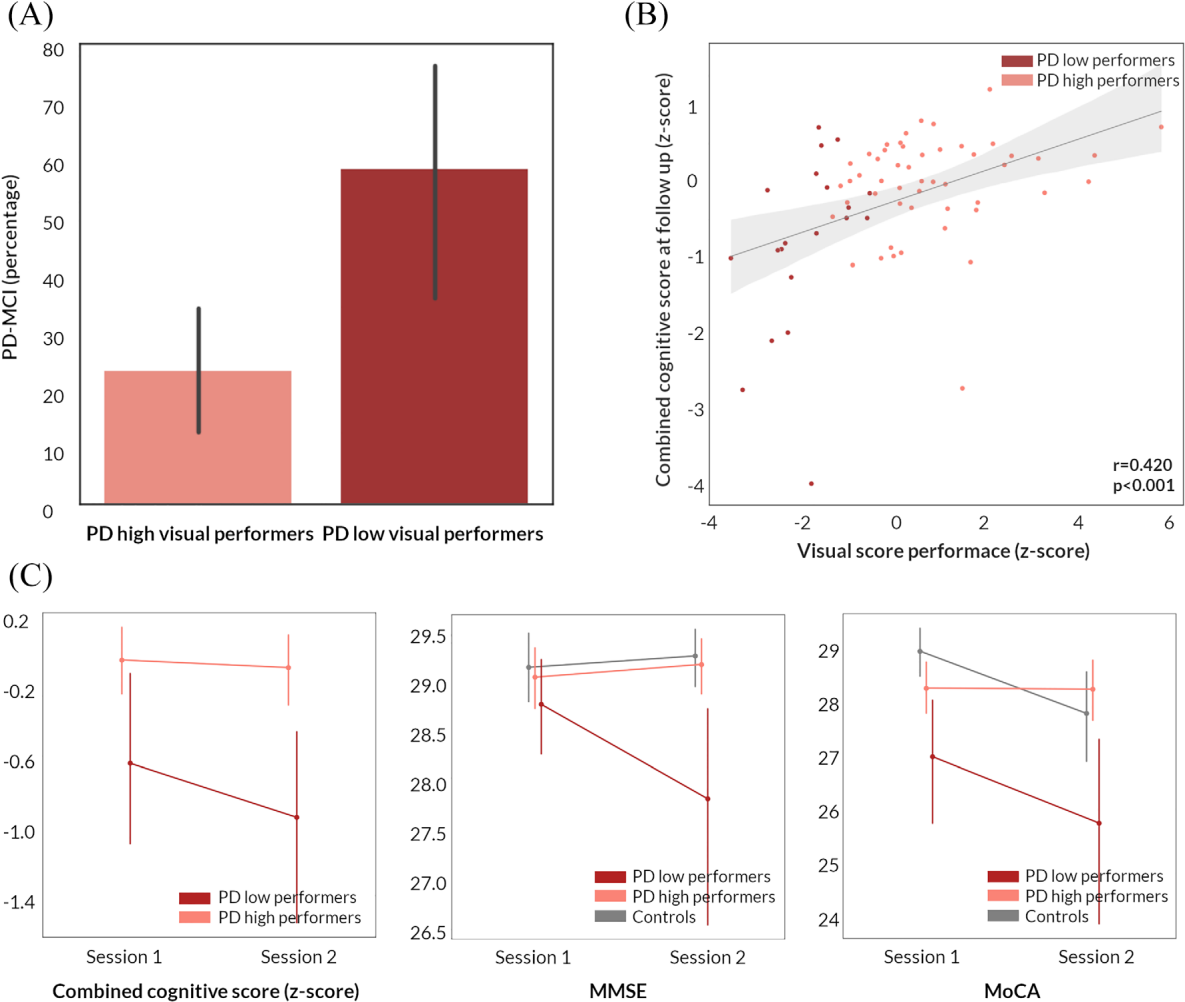
Longitudinal changes in cognition in patients with Parkinson's disease. (**A**) Percentage of patients who developed mild cognitive impairment (PD‐MCI) in PD low visual performers compared with PD high visual performers. (**B**) Correlation between a marker of overall visual performance at baseline and combined cognitive performance at 18‐month follow‐up in patients with Parkinson's disease (95% confidence interval). Visual performance is presented as the summed *z* score of the 2 computer‐based visual tasks (cats and dogs task and biological motion task: *Z* = *Z*_biolmotion_ + *Z*_cats&dogs_). Cognitive performance is presented as combined cognitive score (*z* scored against the performance of age‐matched controls). (**C**) Longitudinal change in combined cognitive score, MMSE and MoCA in PD low visual performers, PD high visual performers, and controls. Error bars represent 95% confidence intervals. MMSE, Mini–Mental State Examination; MoCA, Montreal Cognitive Assessment. [Color figure can be viewed at wileyonlinelibrary.com]

At baseline, visuospatial performance was lower, as expected, in PD low visual performers compared with PD high visual performers and controls (JLO: H = 8.855, *P* = 0.012; and Hooper: H = 20.652, *P* < 0.001). In other cognitive domains, performance at individual cognitive tasks was not significantly different between groups, with the exception of lower performance in PD low visual performers on one test of executive function (Stroop interference: H = 6.642, *P* = 0.036) and one memory task (word recognition task: H = 6.716, *P* = 0.035). Visual performance at computer‐based tasks was correlated with overall cognitive performance at baseline (*r* = −0.306, *P* = 0.011) and at follow‐up (*r* = −0.386, *P* = 0.001); see Figure 1B,C.

Significant changes in longitudinal performance between PD low and PD high visual performers were seen only for MMSE (*t* = −1.084, *P* = 0.024) and letter fluency (*t* = −2.825, *P* = 0.025). However, overall cognitive performance was lower in PD low visual performers, who were more likely to develop MCI at longitudinal follow‐up (χ2 = 7.031, *P* = 0.008; Fig. 1A). Table S2 shows the longitudinal change in cognitive measures in PD low and PD high visual performers.

### 2.2 Whole‐Brain Longitudinal Changes in White Matter Integrity

No significant differences between PD and controls was seen in whole‐brain fixel‐based or voxel‐based analysis, either at baseline or longitudinally.

1. Baseline changes in white matter microstructure and macrostructure related to low visual performance in PD

**FIG. 2:**
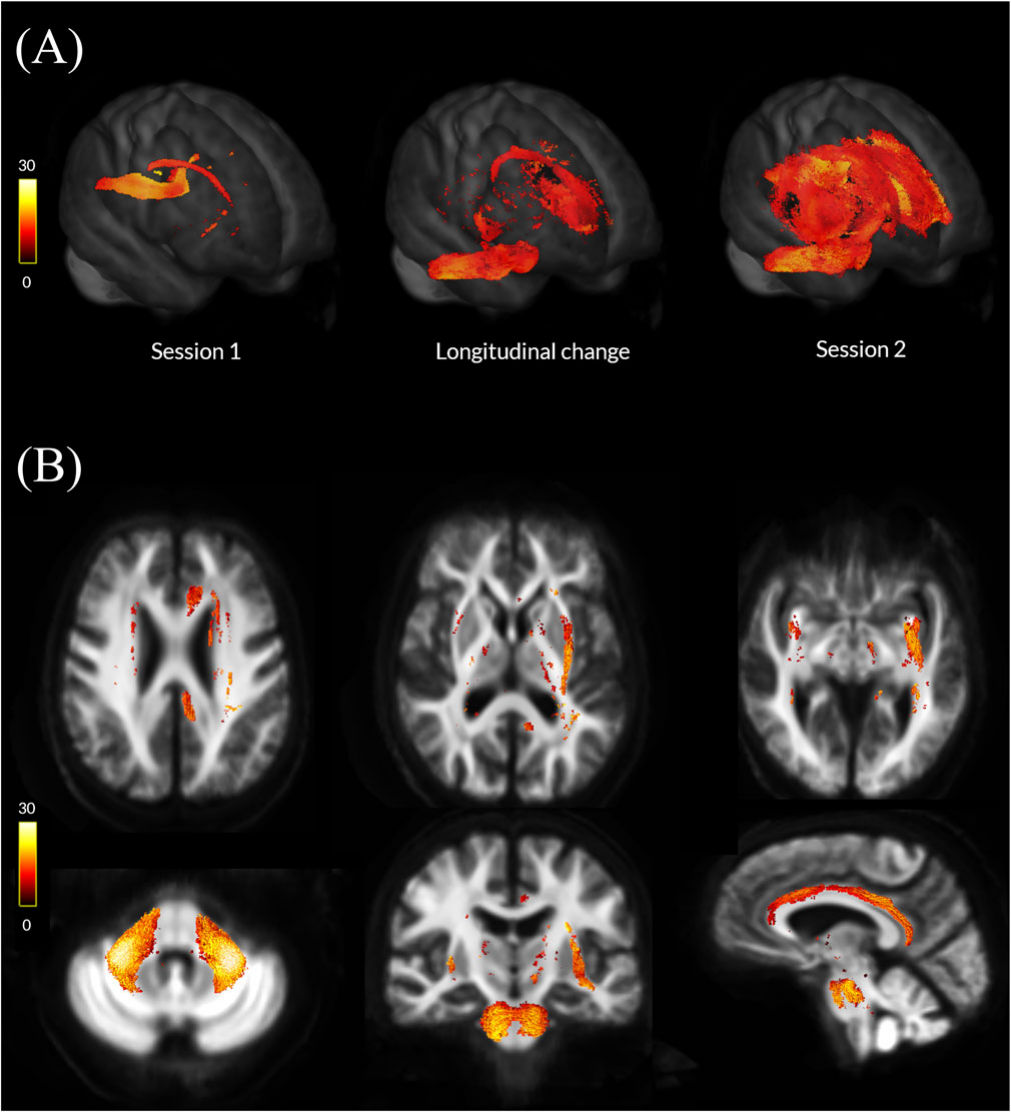
Macrostructural white matter changes in patients with Parkinson's disease and low visual performance over time. (**A**) Changes in white matter macrostructure (as seen by reduction in fiber cross‐section [FC]) in PD low visual performers compared with PD high visual performers (FWE‐corrected *P* < 0.05) in session 1 (baseline, left), at longitudinal change (difference between the 2 images, middle), and in session 2 (18 months follow‐up, right). Results are presented as streamlines and colored by percentage reduction. (**B**) Statistically significant (FWE‐corrected *P* < 0.05) longitudinal reductions in fiber cross‐section (FC) in PD low visual performers compared with PD high visual performers. Results are colored by percentage reduction. [Color figure can be viewed at wileyonlinelibrary.com]

**FIG. 3:**
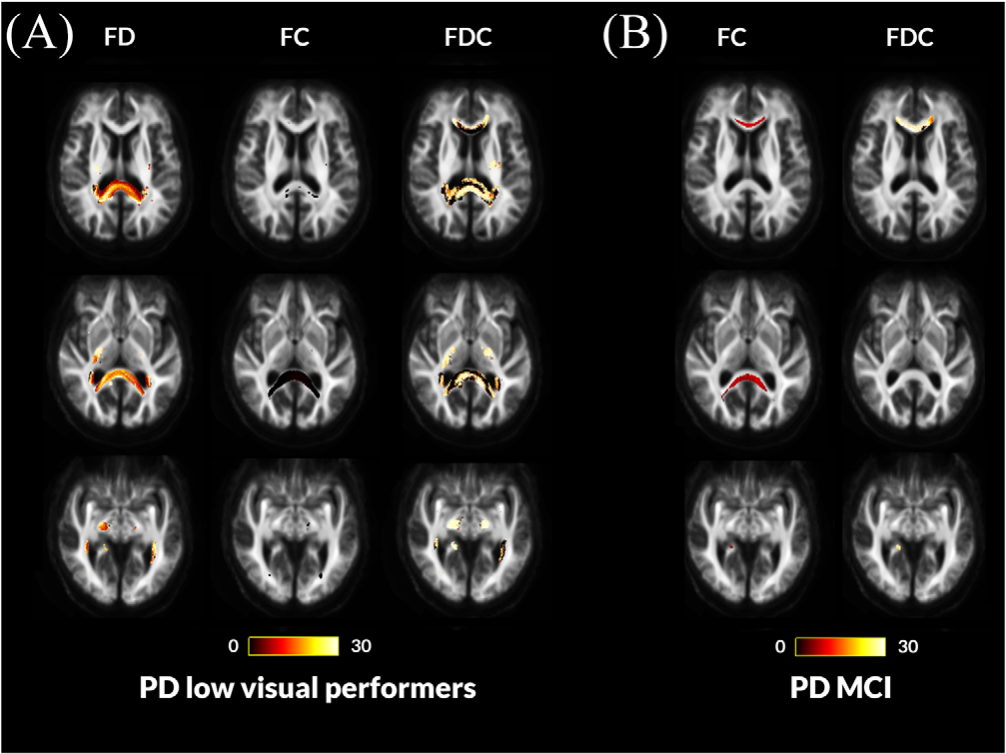
Fiber tract–specific reductions at baseline in PD low visual performers compared with PD high visual performers and PD with mild cognitive impairment compared with PD with normal cognition from whole‐brain fixel‐based analysis. (**A**) PD low visual performers showed widespread microstructural (changes in fiber density [FD]) compared with PD high visual performers, with reductions within the genu, body, and splenium of the corpus callosum, the right internal capsule, the cingulum bilaterally, tapetum bilaterally, posterior thalamic radiations bilaterally, right hippocampus, and the right corticospinal tract. Macrostructural changes (changes in fiber density) were also seen within the splenium of the corpus callosum, the right cingulum, and bilateral posterior thalamic radiations. Changes in the combined FDC metric were seen within the genu, body, and splenium of the corpus callosum, the right internal capsule, the cingulum bilaterally, tapetum bilaterally, posterior thalamic radiations bilaterally, right hippocampus, and the right corticospinal tract; these represent impaired overall ability to relay information in these tracts in PD low visual performers. (**B**) Patients with Parkinson's disease who developed mild cognitive impairment (MCI) showed macrostructural changes (changes in fiber cross‐section [FC]) compared with Parkinson's disease with stable vision within the genu and splenium of the corpus callosum, posterior thalamic radiations bilaterally and the right hippocampus. Changes in the combined FDC metric are seen in the genu and the right hippocampus; this represents impaired overall ability to relay information in these tracts in PD‐MCI compared with PD with normal cognition (NC). No changes were seen in the FD metric for this patient group. Results are displayed as streamlines; these correspond to fixels that significantly differed between PD low and high visual performers (FWE‐corrected *P* < 0.05). Streamlines are colored by percentage reduction (color bars). More details on the baseline changes seen in these groups are seen in Figure S2. [Color figure can be viewed at wileyonlinelibrary.com]

PD low visual performers showed significant changes in white matter macro‐ and microstructure compared with PD high visual performers longitudinally (Fig. 2) and already at baseline (Fig. 3A), as we have shown previously.^13^ At baseline, PD low visual performers showed reduction in FC within the splenium of the corpus callosum, right cingulum, and bilateral posterior thalamic radiations. Extensive microstructural changes (FD reductions) were also present at baseline in PD low visual performers within the genu, body, and splenium of the corpus callosum, right internal capsule, cingulum bilaterally, tapetum bilaterally, posterior thalamic radiations bilaterally, right hippocampus, and right corticospinal tract. Reductions were seen across the same regions in the combined FDC metric; these were particularly pronounced within the genu and splenium of the corpus callosum, with more than a 30% reductions in FDC. Figure 3A illustrates the extent of baseline macro‐ and microstructural changes in PD low visual performers.

1. White matter macrostructural changes in patients with PD‐MCI

MCI status was less sensitive than visual performance in identifying baseline white matter alterations in PD, with fewer tracts affected. PD‐MCI patients (including those with normal baseline cognition who later converted to PD‐MCI) showed reductions in FC within the genu and splenium of the corpus callosum, right posterior thalamic radiation, and right hippocampus (Fig. 3B). Although there were no statistically significant changes in FD, the combined FDC metric showed significant, more than 30%, reductions in the genu, and right hippocampus (Fig. 3B). Given that white matter changes in PD‐MCI and PD‐low visual performers had differential spatial profiles, direct comparison is difficult. However, in both cases, the splenium of the corpus callosum was the most affected tract. In direct comparison of mean baseline FD, FC and FDC of the splenium, the area under the curve was 0.615 for PD‐MCI and 0.673 in PD‐low visual performers, suggesting slightly higher sensitivity of visual performance for white matter alterations even within the same tract.

1. Longitudinal white matter alterations are seen in patients with PD and low visual performance

PD low visual performers showed significant changes in white matter macrostructure, with up to 22% reductions in FC compared with PD high visual performers in whole‐brain fixel‐based analysis. These changes were extensive, involving multiple tracts corrected for age, sex, and intracranial volume, including the middle cerebellar peduncles pontine crossing parts and external capsules bilaterally, left inferior and superior fronto‐occipital fasciculi, uncinate fasciculi bilaterally, left cingulum, left anterior and posterior corona radiata, and genu (Fig. 2). Importantly, cognitive measures, including MoCA and MCI status (both assessing MCI at baseline and at follow‐up together, and in separate subassessments within each group), and 2 clinical risk scores for dementia^53^, ^54^ were not associated with longitudinal white matter micro‐ or macrostructural changes.

### 2.3 Voxel‐Based Analysis

Conventional voxel‐based analysis did not show any statistically significant differences after FWE‐correction for PD versus controls or PD‐MCI versus PD‐NC at baseline or longitudinal follow‐up. PD low visual performers did not show any statistically significant differences at baseline. At longitudinal imaging, PD low visual performers showed reduced FA within the left external capsule and right posterior thalamic radiation and increased MD within the splenium of the corpus callosum.

### 2.4 Tract of Interest Analysis

**FIG. 4:**
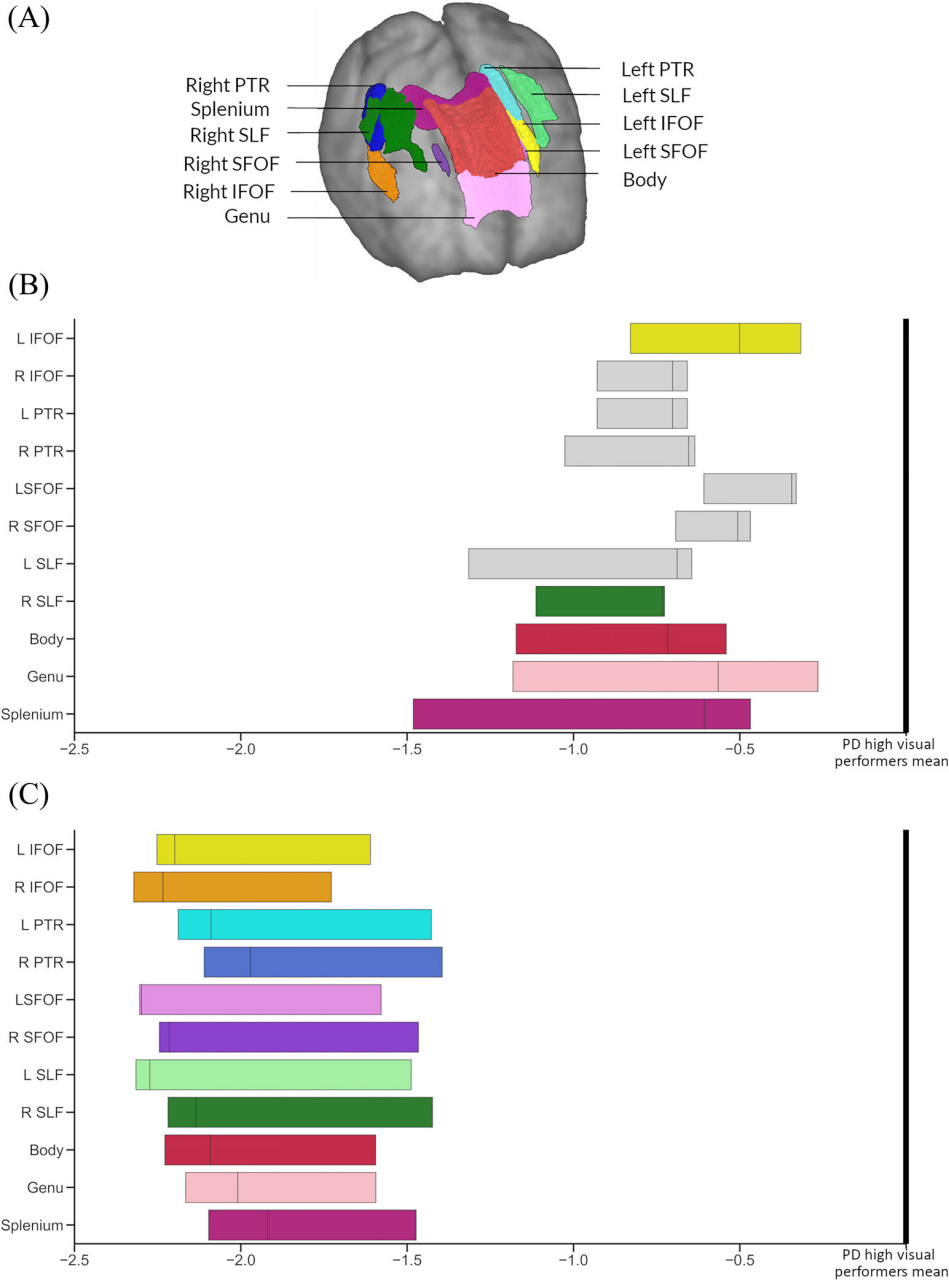
Significant tracts in Parkinson's low performers; tract of interest analysis. (**A**) Anatomical representation of all analyzed tracts. PTR, posterior thalamic and optic radiations; SLF, superior longitudinal fasciculi; IFOF, Inferior fronto‐occipital fasciculi (segmentation includes the inferior longitudinal fasciculus); and SLF, superior fronto‐occipital fasciculi. (**B**) Baseline visit. Reduction (mean, 95% CI) in fiber cross‐section (FC) visualized as percentage reduction from the mean of patients with Parkinson's disease with high visual performance. Tracts with significantly reduced FC (FDR‐corrected *P* < 0.05) are shown in color, whereas tracts with no significant changes in FDC are plotted in gray. L, left; R, right; **C**, visit 2 (18‐month follow‐up). Reduction (mean, 95% CI) in fiber cross‐section (FC) visualized as percentage reduction from the mean of patients with Parkinson's disease with high visual performance at follow‐up. All 11 of the selected tracts showed significantly reduced FC (FDR‐corrected *P* < 0.05). L, left; R, right. [Color figure can be viewed at wileyonlinelibrary.com]

Widespread reductions were seen in mean FC in PD low visual performers compared with PD high visual performers in tract‐of‐interest analysis. Specifically, at baseline after correction for age, sex, and intracranial volume, significant changes were seen across the corpus callosum (genu: *t* = −0.086, FDR‐corrected q = 0.002; body: *t* = −0.076, q = 0.002; splenium: *t* = −0.099, q = 0.002), left inferior fronto‐occipital fasciculus (*t* = −0.037, q = 0.002) and right superior longitudinal fasciculus (*t* = −0.027, q = 0.002); see Figure 4B. At longitudinal follow‐up, all 11 tracts showed significant reductions (FDR‐corrected) in PD low visual performers compared with high visual performers (Fig. 4C).

Across patients with PD, higher reductions in mean FC across the 11 selected tracts were associated with worsening MoCA scores (*r* = 0.258, *P* = 0.024) but not MMSE scores at 18‐month follow‐up.

Results were unchanged when including total years spent in education as a covariate (in addition to age, sex, and total intracranial volume) in both whole‐brain and tract‐of‐interest analyses.

## 3 Discussion

Low visual performance in PD was associated with cognitive decline and widespread white matter macrostructural changes at the 18‐month follow‐up. Specifically, baseline low visual performance was associated with: (1) worsening cognition and conversion to MCI at the 18‐month follow‐up, (2) further reductions in fiber cross‐section at longitudinal follow‐up, and (3) more extensive white matter changes at baseline than those seen in those patients who developed mild cognitive impairment. These findings provide evidence that visual changes in PD are a marker for incipient white matter degeneration and cognitive decline.

### 3.1 White Matter Alterations Precede Cognitive Impairment in Parkinson's Disease

We have previously described the white matter changes associated with low visual performance at baseline.^13^ We found similar but significantly less extensive white matter changes in patients who later progressed to develop MCI, specifically involving the right hippocampus and the genu and splenium of the corpus callosum. Changes within the corpus callosum, particularly the most anterior and most posterior segments, have been reported in PD with cognitive impairment using diffusion tensor imaging.^10^, ^58^, ^59^, ^60^, ^61^ Similar changes were seen using fixel‐based analysis in association with disease severity, particularly nonmotor symptoms,^14^ and in patients with PD and poor visual performance.^13^

The hippocampus has a higher Lewy body count and greater cholinergic deficit in PD dementia,^62^ but studies assessing gray matter atrophy and neurotransmitter changes in vivo in PD with cognitive impairment have also demonstrated changes outside the hippocampus,^63^, ^64^, ^65^ leading to debate regarding the relative importance of the hippocampus in PD dementia. In a prior meta‐analysis using network‐lesion mapping, our group has shown that hippocampal networks, particularly of the right hippocampus, which plays a crucial role in spatial memory,^66^ are linked to PD dementia.^67^ Our finding of right hippocampal tract involvement in PD‐MCI provides corroborative evidence that hippocampal networks are implicated in the development of PD dementia. Gray matter atrophy and white matter degeneration are closely linked, and establishing how they interact in the development of cognitive impairment in PD is difficult in vivo. New emergent compartment‐based methods that can differentiate between the soma and neurite might be able to better assess this in the future.^68^

In patients with low visual performance, in addition to more extensive alterations of both white matter microstructure and morphology at baseline, additional macrostructural changes developed at follow‐up. The right hippocampus was also affected in PD low visual performers together with its temporal lobe connections, such as the cingulum and inferior longitudinal fasciculus, which have been implicated in Alzheimer's disease as well as Parkinson's disease with cognitive impairment.^69^

Long corticocortical tracts connecting frontal to occipital regions, such as the superior and inferior fronto‐occipital fasciculi, and subcortical–cortical tracts, such as the external capsule, became bilaterally affected at follow‐up (particularly in the left hemisphere). The fronto‐occipital fasciculi play a role in global cognition, attention, visual processing, and executive function.^70^, ^71^ The external capsule acts as a route for cholinergic pathways, and diffusion‐derived metrics have been associated with cognitive performance in healthy older adults.^72^ Long‐range connections play a crucial role in brain integration at the network level,^73^ and functional connectivity is reduced for fronto‐occipital connections in PD with cognitive impairment.^74^, ^75^

The cerebellar tracts, particularly the middle cerebellar peduncles, showed significant macrostructural changes at follow‐up in PD low visual performers. The corticopontocerebellar pathway, which connects cortical regions to the cerebellum via the contralateral middle cerebellar peduncle, may play a key role in cognition.^76^, ^77^ In patients with Parkinson's disease, functional connectivity between the vermis and visual association and prefrontal cortex is weaker than in controls and correlates with cognitive performance, even in the absence of cerebellar volume loss or changes in cortical thickness.^41^ Although traditionally regarded as unaffected by α‐synuclein pathology, recent evidence suggests that cerebellar nuclei and surrounding white matter do accumulate α‐synuclein aggregates.^78^ Our findings provide further support for structural white matter changes within the cerebellum in patients with PD and cognitive impairment.

In patients with PD and poor visual performance, we saw widespread white matter alterations, with interhemispheric connections in the corpus callosum, with long fronto‐occipital and hippocampal connections affected first, followed by diffuse macrostructural changes across multiple association tracts. These diffuse changes may explain the alterations in global functional and structural connectivity seen in PD^79^, ^80^, ^81^ and emphasize the new insights that network approaches can provide in our understanding of Parkinson's dementia.

There was no difference in visual acuity between patients classified as high versus low visual performers based on the higher‐order visual tests, although we did find worse contrast sensitivity in low visual performers. Contrast sensitivity is affected by ophthalmic disease (including retinal disease) as well as thalamic and cortical dysfunction, suggesting some contribution from anterior pathways. We have previously shown that visual dysfunction from ophthalmic as well as higher‐order pathways is linked with higher risk of dementia in PD.^5^ This is supported by recent population‐based studies showing that visual dysfunction, including from ophthalmic causes, is related to poorer outcomes in PD.^4^, ^82^

### 3.2 Impending Cognitive Decline and Macrostructural White Matter Degeneration in Patients With PD and Visual Dysfunction: An Opportunity for Intervention

Baseline visual performance was associated with cognitive performance at follow‐up in patients with PD, and poor visual performance predicted worsening cognition and development of MCI. Prior work from our group has shown that visuoperceptual deficits are linked to poorer cognitive performance at baseline and clinical scores for dementia^5^, ^16^ and worsening MoCA at the 1‐year follow‐up.^18^ This study provides further evidence of impending cognitive impairment in patients with PD and visuoperceptual deficits.

We also found that PD low visual performers exhibited significant and widespread reduction in fiber cross‐section, signaling widespread macrostructural white matter alterations at 18 months. In contrast, patients with PD who were classified as high dementia risk by 2 clinically‐derived scores^53,53^ and those who developed MCI at follow‐up did not exhibit further white matter alterations, with PD‐MCI showing established white matter degeneration at baseline. These findings raise the possibility that poor visual performance could represent a window of opportunity for therapeutic interventions in patients with PD by identifying patients with imminent but not yet clinically apparent cognitive impairment and white matter degeneration.

### 3.3 Limitations and Future Directions

Baseline PD‐MCI rates were lower than the ~40% incidence previously reported in clinic‐based studies,^83^ likely to represent selection bias in recruitment because of our study exclusion criteria, which included a diagnosis of PD dementia and higher rates of participation in research among cognitively intact individuals. Indeed, conversion rates at the 18‐month follow‐up were 20.6% (13 of the 67 patients with PD who were cognitively intact at baseline), comparable to other published studies.^84^ Our study was not able to assess the potential impact of coexistent cerebrovascular disease in PD‐associated cognitive impairment as: (1) only participants without a history of neurological conditions were included in the study, (2) because of the acquired MRI sequences, we could not systematically quantify white matter hyperintensities, and (3) although we asked for a self‐reported history of vascular risk factors, we have not assessed these biochemically. Although there were no significant group differences in baseline vascular risk factors in our cohort, and all raw imaging data were visually inspected to exclude clinically significant cerebrovascular disease burden, further studies are needed to assess how cerebrovascular disease influences the temporal order of cognitive impairment in Parkinson's disease, given that it contributes to cognitive decline in the presence of neurodegeneration.^85^ In addition, white matter hyperintensities could affect fixel‐based metrics, specifically leading to fiber density reduction.^86^ Because of the acquired MRI sequences, we could not systematically quantify and control for white matter hyperintensities; this is in line with other fixel‐based analysis studies,^14^, ^56^, ^87^, ^88^ but future studies need to clarify the exact effect of white matter hyperintensities on fixel‐based metrics. At both times, participants with PD underwent imaging on their usual dopaminergic medications. Corrected fractional anisotropy is not affected by levodopa^89^; therefore, it is unlikely that dopaminergic medication would affect fixel‐based metrics. In addition, levodopa‐equivalent doses did not significantly differ between PD low and high visual performers at any time. Follow‐up imaging and psychological testing were performed at 18 months; longer follow‐up of patients with PD who will proceed to develop dementia could provide further insights into the temporal order of white matter degeneration in PD with cognitive impairment.

## 4 Conclusions

Poor visual function in Parkinson's disease is associated with worsening cognition and conversion to longitudinal white matter macrostructural changes. These findings provide insights into the temporal pattern of white matter degeneration associated with cognitive impairment in Parkinson's disease and highlight an at‐risk population for possible therapeutic intervention.

## Author Contributions

Study design and concept: A.Z., R.W.; data collection: A.Z., LA.L.; R.W.; imaging and statistical analysis: A.Z.; drafting and revision of the manuscript: A.Z., P.M.C., L.A.L., A.L., R.W.

## Supporting information


**Appendix S1**. Supplementary Information
