# Supplementary material for: Visual Dysfunction Predicts Cognitive Impairment and White Matter Degeneration in Parkinson's Disease
Source: Mov Disord. 2021 Jan 9;36(5):1191–202. doi: 10.1002/mds.28477 (PMC8248368; doi:10.1002/mds.28477)
Supplement: Supplementary file 1 — Appendix S1. Supplementary Information [file MDS-36-1191-s001.docx]

**Supplementary Material for:**

**Visual dysfunction predicts cognitive impairment and white matter degeneration in Parkinson’s disease**

Angeliki Zarkali^1^, Peter McColgan^2^, Louise-Ann Leyland^1^, Andrew J. Lees^3^, Rimona S. Weil^1,4,5^

Contents

[**Supplementary Figure 1: Examples of the computer-based visual stimuli** 2](#_Toc55311939)

[**Supplementary Table 1. Cerebrovascular risk factors in our cohort (self-reported)** 4](#_Toc55311940)

[**Supplementary Table 2. Longitudinal cognitive changes in patients with Parkinson’s disease.** 5](#_Toc55311941)

[**Supplementary Figure 2. Baseline white matter changes in patients with Parkinson’s disease.** 6](#_Toc55311942)

# **Supplementary Figure 1: Examples of the computer-based visual stimuli**


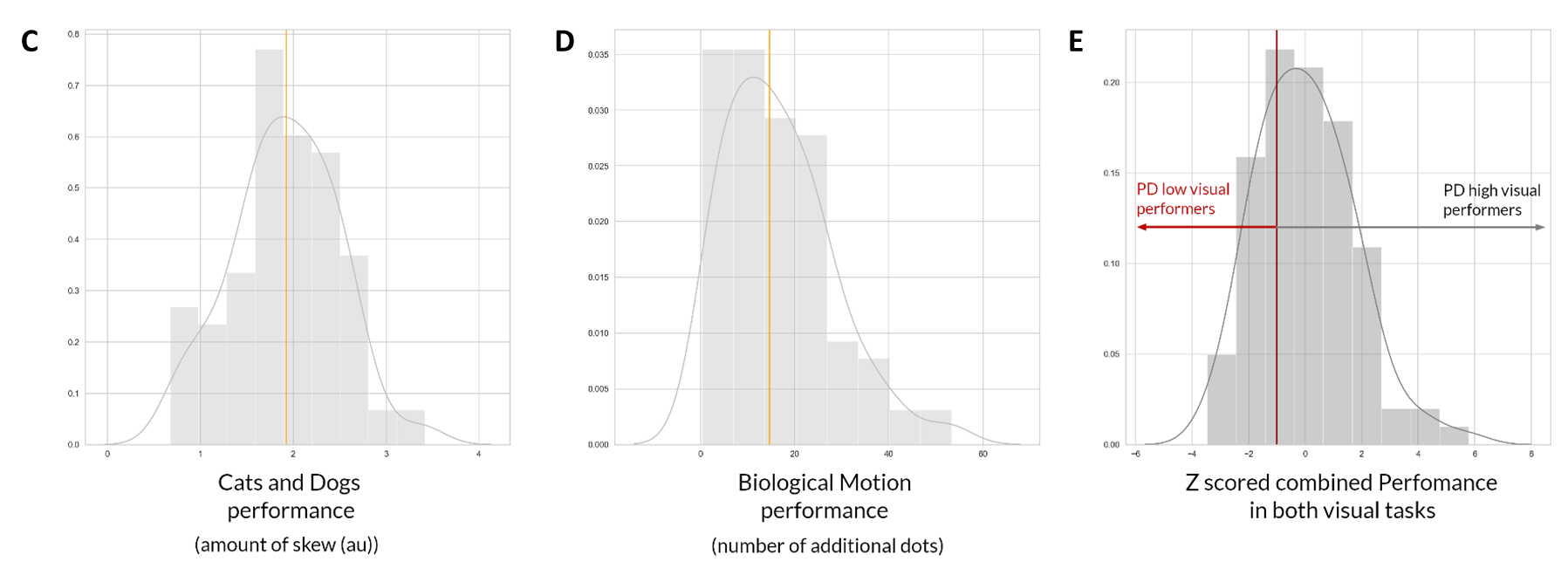
**
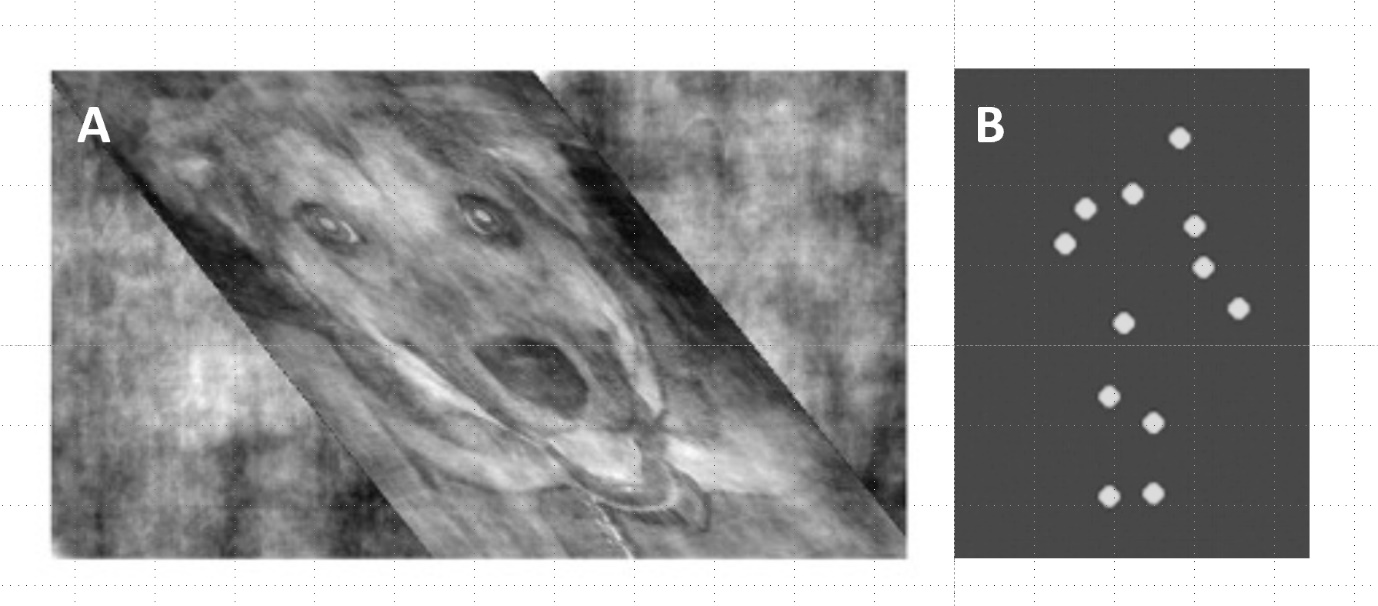
**

Participants with PD were classified according to their performance in two computer-based higher-order visual tasks. The Cats and Dogs task (***A***: example stimulus) measures tolerance to visual skew, with images of cats and dogs distorted by varying skew along the x-axis and threshold of visual skew determined using psychophysical testing (two alternative forced choice, 90 repetitions, presentation time: 280ms, increasing visual skew applied) as described previously^4,7,43^.

The biological motion task (***B***: example frame from an amination depicting a person walking) measures sensitivity to perception of a moving person from moving dots at the position of the major joints. Increasing the number of moving dots makes the task more difficult, and the number of additional dots tolerated is determined psychophysically, as previously described^44^. These visual tasks were chosen as they provide robust measures of higher order visual function and have been shown by our group to be associated with higher risk of PD dementia and worsening cognition over time^4,7,44^.

The performance of PD participants in the two experimental tasks is seen in panel ***C*** for the Cats and Dogs task and ***D*** for the Biological motion task, with the orange line highlighting the median performance in each task.

To capture patients with consistently poor performance in both high-level visual tasks, we classified patients as poor visual performers if they performed worse than the group median in both tasks (individual Cats and Dogs Performance less than median performance in the Cats and Dogs task & individual Biological Motion Performance less than median performance in the Biological Motion task). This led to 33 patients with PD classified as low visual performers. All other patients with PD were classified as high visual performers (n=58) as in previous work^4,79,80^. 30 unaffected age-matched controls were recruited from spouses and a volunteer database; controls were matched to the PD group as whole.

In addition to this classification, we calculated a combined visual performance score. As the two visual tasks have different score scales, we used Z scored performance in each score (compared to the average control-group performance) to calculate this (Z_score_ = Z_Biolmotion_ + Z_cats&dogs_).

# **Supplementary Table 1. Cerebrovascular risk factors in our cohort (self-reported)**

| **History of:** | **Controls**  **n= 25** | **PD high visual performers**  **n= 54** | **PD low visual performers**  **n= 22** | **Statistic** |
| --- | --- | --- | --- | --- |
| **Previous stroke / transient ischaemic attack** | 0 (0) | 1 (1.9) | 2 (9.1) | x^2^=3.920  p=0.140 |
| **Angina** | 0 (0) | 0 (0) | 1 (4.5) | x^2^=3.672  p=0.159 |
| **Hypertension** | 5 (20.0)) | 6 (11.1) | 6 (27.3) | x^2^=3.105  p=0.212 |
| **Diabetes** | 0 (0) | 1 (1.9) | 2 (9.1) | x^2^=3.927  p=0.140 |
| **Hypercholesterolaemia** | 8 (32.0) | 6 (11.1) | 6 (27.3) | x^2^=5.348  p=0.069 |

# **Supplementary Table 2. Longitudinal cognitive changes in patients with Parkinson’s disease.**

| **Cognitive test** | **PD high visual performers**  **n= 55** | **PD low visual performers**  **n= 22** | **PD high visual performers**  **n= 55** | **PD low visual performers**  **n= 22** | **Statistic** |
| --- | --- | --- | --- | --- | --- |
| ***General cognition*** | **Baseline visit** | | **Follow up visit**  **(18 months)** | | **p-value*** |
| MOCA | 28.3 (1.9) | 27.0 (2.7) | 28.2 (2.1) | 25.8 (4.2) | 0.113 |
| MMSE | **29.1 (1.7)** | **28.8 (1.2)** | **29.2 (1.0)** | **27.9 (2.6)** | **0.024** |
| ***Attention*** |  |  |  |  |  |
| Digit span forwards | 9.4 (1.8) | 8.9 (2.4) | 9.9 (1.6) | 9.5 (2.2) | 0.601 |
| Digit span backwards | 7.5 (2.1) | 6.9 (2.4) | 8.0 (2.3) | 7.4 (2.6) | 0.623 |
| Stroop: Colour (sec) | 32.7 (6.7) | 36.4 (7.8) | 33.2 (6.8) | 39.3 (15.2) | 0.246 |
| ***Executive function*** |  |  |  |  |  |
| Stroop: Interference (sec) | 58.9 (16.9) | 73.1 (28.5) | 56.2 (17.1) | 74.3 (29.9) | 0.386 |
| Category fluency | 22.3 (5.1) | 20.4 (7.1) | 21.4 (4.8) | 16.8 (6.7) | 0.068 |
| ***Memory*** |  |  |  |  |  |
| Word Recognition Task | 24.4 (1.3) | 23.7 (1.3) | 24.2 (1.3) | 23.7 (1.3) | 0.109 |
| Logical Memory (delayed) | 13.8 (3.9) | 11.9 (5.6) | 12.2 (3.0) | 10.2 (3.7) | 0.899 |
| ***Language*** |  |  |  |  |  |
| Graded Naming Task | 24.3 (2.4) | 23.6 (2.9) | 24.9 (2.9) | 23.5 (3.8) | 0.322 |
| Letter Fluency | **16.7 (4.5)** | **17.9 (6.7)** | **17.0 (4.6)** | **15.5 (5.2)** | **0.025** |
| ***Visuospatial*** |  |  |  |  |  |
| JLO | 25.7 (3.5) | 22.7 (4.3) | 25.7 (3.4) | 22.2 (4.8) | 0.738 |
| Hooper | 25.7 (2.5) | 22.6 (2.8) | 25.8 (2.9) | 22.2 (4.1) | 0.622 |
| ***Hallucinations*** |  |  |  |  |  |
| Habitual hallucinations | 6 (11.1) | 6 (27.3) | 8 (14.5) | 7 (31.8) | p=0.170 |
| UM-PDHQ | 0.5 (1.8) | 1.1 (2.1) | 0.5 (1.5) | 1.3 (3.0) | p=0.145 |
| *All data shown are mean (SD), except habitual hallucinations which is shown as number of participants (%).*  ** Statistical comparison of individual performance change (Performance in Follow up visit – Performance in Baseline visit) for each metric; using t test for normally distributed variables and Kruskal wallis for non-normally distributed variables. In bold characteristics that significantly differed in terms of change between Visit 2 and baseline between groups.*  *JLO: Judgement of Line Orientation. UM-PDHQ: University of Miami Parkinson’s Disease hallucinations questionnaire: Higher score indicate more severe hallucinations.* | | | | | |

# **Supplementary Figure 2. Baseline white matter changes in patients with Parkinson’s disease.**


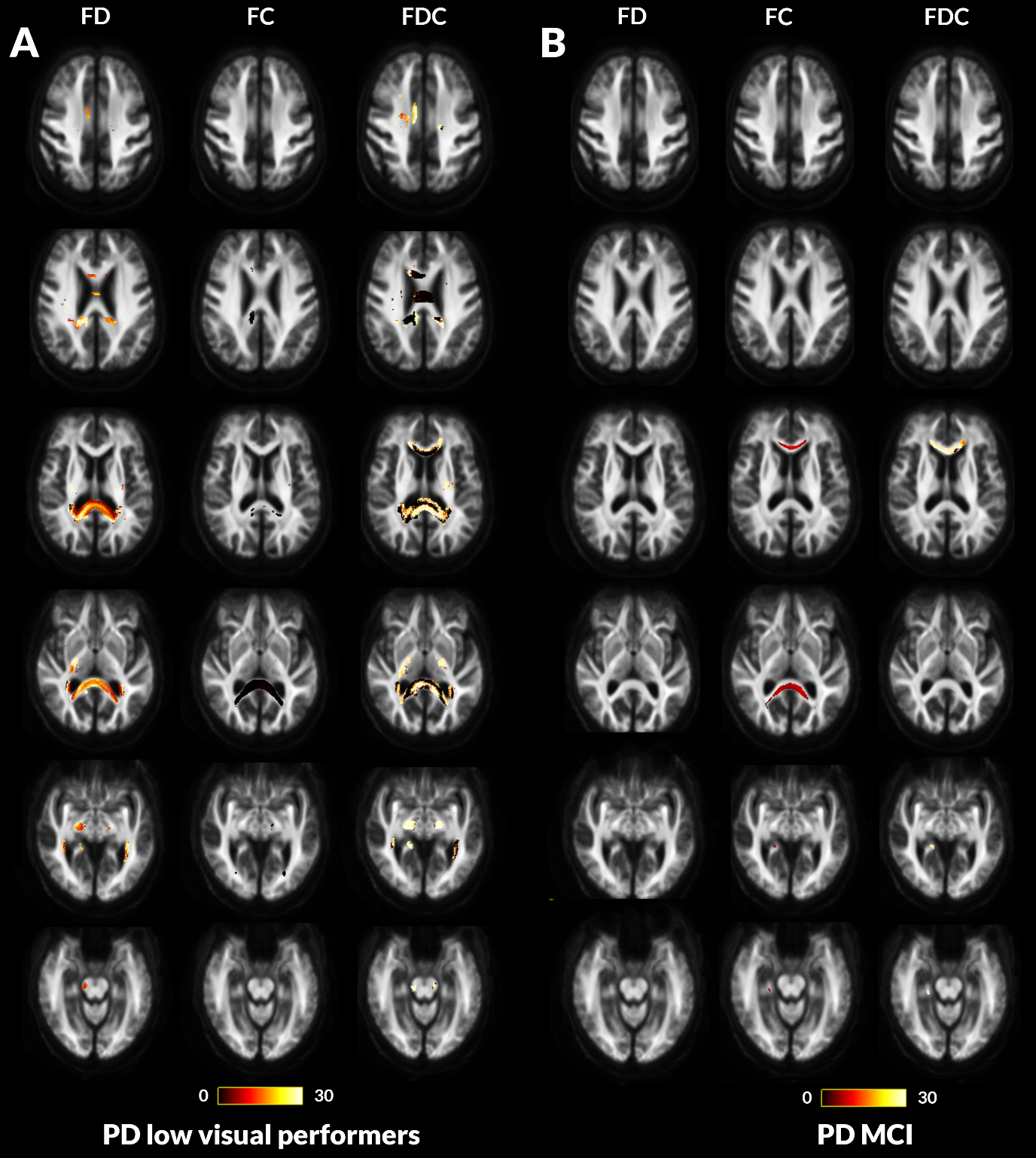


Fibre tract-specific reductions at baseline in PD low visual performers compared to PD high visual performers and PD with mild cognitive impairment compared to PD with normal cognition from whole-brain fixel-based analysis.

A. PD low visual performers showed widespread microstructural (changes in fibre density (FD)) compared to PD high visual performers, with reductions within the genu, body and splenium of the corpus callosum, the right internal capsule, the cingulum bilaterally, tapetum bilaterally, posterior thalamic radiations bilaterally, right hippocampus and the right corticospinal tract. Macrostructural changes (changes in fibre density (FC) were also seen within the splenium of the corpus callosum, the right cingulum and bilateral posterior thalamic radiations. Changes in the combined FDC metric were seen within the genu, body and splenium of the corpus callosum, the right internal capsule, the cingulum bilaterally, tapetum bilaterally, posterior thalamic radiations bilaterally, right hippocampus and the right corticospinal tract; these represent impaired overall ability to relay information in these tracts in PD low visual performers.

B. Patients with Parkinson’s disease who developed mild cognitive impairment (MCI) showed macrostructural changes (changes in fibre cross-section (FC)) compared to Parkinson’s disease with stable vision within the genu and splenium of the corpus callosum, posterior thalamic radiations bilaterally and the right hippocampus. Changes in the combined FDC metric are seen in the genu, and the right hippocampus; this represents impaired overall ability to relay information in these tracts in PD-MCI compared to PD with normal cognition (NC). No changes were seen in the FD metric for this patient group.

Results are displayed as streamlines; these correspond to fixels that significantly differed between PD low and high visual performers (FWE-corrected p <0.05). Streamlines are coloured by percentage reduction (colourbars).
